# Tetrahedral DNA Framework-Programmed Electrochemical Biosenors with Gold Nanoparticles for Ultrasensitive Cell-Free DNA Detection

**DOI:** 10.3390/nano12040666

**Published:** 2022-02-16

**Authors:** Chenguang Wang, Wei Wang, Yi Xu, Xiaoshuang Zhao, Shuainan Li, Qiuling Qian, Xianqiang Mi

**Affiliations:** 1 Shanghai Advanced Research Institute, Chinese Academy of Sciences, Shanghai 201210, China; wangcg@sari.ac.cn (C.W.); xuyi@sari.ac.cn (Y.X.); lishuainan2019@sari.ac.cn (S.L.); qianqiuling2018@sari.ac.cn (Q.Q.); 2 University of Chinese Academy of Sciences, Beijing 100049, China; 3 Shanghai Pudong New District Zhoupu Hospital, Shanghai University of Medicine & Health Sciences Affiliated Zhoupu Hospital, Shanghai 201318, China; doctorww1978@163.com; 4 State Key Laboratory of Functional Material for Informatics, Shanghai Institute of Microsystem and Information Technology, Chinese Academy of Sciences, Shanghai 200050, China; zxsh1217@mail.sim.ac.cn; 5 CAS Center for Excellence in Superconducting Electronics (CENSE), Shanghai 200050, China; 6 Key Laboratory of Systems Health Science of Zhejiang Province, Hangzhou Institute for Advanced Study, University of Chinese Academy of Sciences, Chinese Academy of Sciences, Hangzhou 310024, China

**Keywords:** tetrahedral DNA framework, gold nanoparticles, hybridization chain reaction, cell-free DNA, electrochemical biosenors

## Abstract

Tumor-associated cell-free DNA (cfDNA) is a dynamic biomarker for genetic analysis, early diagnosis and clinical treatment of cancers. However, its detection has limitations because of its low abundance in blood or other complex bodily fluids. Herein, we developed an ultrasensitive cfDNA electrochemical biosensor (E-cfDNA sensor) based on tetrahedral DNA framework (TDF)-modified gold nanoparticles (Au NPs) with an interface for cfDNA detection. By accurately controlling the numbers of base pairs on each DNA framework, three types of TDFs were programmed: 26 base pairs of TDF; 17 base pairs of TDF; and 7 base pairs of TDF (TDF-26, TDF-16 and TDF-7, respectively). We also combined the TDF with hybridization chain reaction (HCR) to achieve signal amplification. Under optimal conditions, we detected the breast cancer susceptibility gene 1 (BRCA-1), a representative cfDNA closely related to breast cancer. An ultra-low detection limit of 1 aM with a linear range from 1 aM to 1 pM by TDF-26 was obtained, which was superior to the existing methods. Each type of TDF has excellent discrimination ability, which can distinguish single mismatch. More significantly, we also detected BRCA-1 in mimic serum samples, demonstrating that the E-cfDNA sensor has potential use in clinical research.

## 1. Introduction

Tumor-associated cell-free DNA (cfDNA) is a dynamic biomarker derived from different release mechanisms, such as necrosis, apoptosis and active release from carcinoma cells [1,2]. cfDNA can be used for early cancer diagnosis, gene mutation diagnosis, assisted targeted therapy and prognosis, etc. [3,4]. However, due to its extremely low content in blood or other bodily fluids, it is necessary to develop an ultrasensitive detection system for the analysis of cfDNA.

In recent decades, a certain number of technologies have been reported for the analysis of cfDNA, including polymerase chain reaction, DNA sequencing [5,6], microarray [7], nanomaterial-based biosensors [8,9], etc. Among these technologies, electrochemical-based biosensors are regarded as a promising direction for cfDNA detection because of their advantages of high sensitivity, low cost, and miniaturization [10,11,12]. In order to improve the detection performance of electrochemical analysis, quite a few methods have been studied. For example, as an important branch of electrochemical biosensors, screen-printed carbon electrodes (SPCE) are not only reproducible and inexpensive, but can also be prepared into multi-channel electrodes to achieve high-throughput detection and improve detection efficiency [13]. However, the screen-printed carbon surface is highly rough and prone to nonspecific adsorption [14]. Inorganic nanomaterials such as gold nanoparticles (AuNPs) can be modified on the electrode surface, providing excellent electrical conductivity, high surface area to volume ratio, superior catalytic capability and stability, and the ability to control the electrode microenvironment [15,16,17].

AuNPs are used to boost the fixation of the capture probe through the Au-S bond [18,19]. The traditional capture probes are usually thiolated single-stranded DNA [20]. Nevertheless, it is hard to modulate the density and orientation of the capture probes at the interface, which affect their recognition and binding of target molecules [21]. In recent years, tetrahedral DNA framework (TDF), a kind of three-dimensional programmable soft lithography nanomaterial, has been used as a capture probe to be fixed on the surface of the gold electrode [22]. Due to its rigid structure and controllable size, the TDF could precisely control the direction of the capture probes and the distance between the probes, avoiding molecular entanglements, providing a solution-phase-like environment, and improving the sensitivity of electrochemical sensors [23,24,25]. Lin et al. used millimeter-sized gold electrodes modified with different sizes of TDFs for DNA detection, which achieved attomolar sensitivity [26]. Our group has successfully combined TDFs with poly-adenine-based AuNPs for the analysis of BRCA1 with a detection limit of 0.1 fM [27].

In order to further improve the detection performance of electrochemical cfDNA biosensors, various signal amplification strategies are introduced into the biosensors, such as enzyme amplification strategies [28,29], and nanoparticle-based amplification strategies [30,31]. The hybridization chain reaction (HCR) is a toehold-mediated amplification reaction that could be applied to solid-state interfaces, such as electrodes, nanoparticles, glass slides or microfluidic chips, etc. [32]. The HCR products combine with other output moleculars to achieve the ultrasensitive detection of nucleic acid. Yang et al. reported an HCR-based electrochemical genosensor, in which the capture probe was immobilized on the electrode substrate through Au-S bonds, realizing BRCA1 detection with ultrahigh sensitivity [33]. Ge et al. developed an electrochemical biosensor based on the synergistic effect of the TDF and the HCR. TDF could control the density and direction of the capture probes and targets, while the targets triggered the HCR that combined with HRP to achieve sensitive detection of miRNAs [34].

Herein, given the synergistic superiority of TDF and HCR, we exploited home-made Au NPs modified with a multichannel electrochemical biosensor for ultrasensitive detection of cell-free DNA (E-cfDNA sensor). Using this platform, we detected the breast cancer susceptibility gene 1 (BRCA1, a representative cfDNA closely related to breast cancer) that achieved a detection limit at the attomolar level. In addition, this E-cfDNA sensor also realized the discrimination of a single-base mismatch. Moreover, this platform provides a universal tool for other cfDNA, and has great potential in clinical liquid biopsy.

## 2. Materials and Methods

### 2.1. Materials and Instruments

All nucleotide sequences used (Table 1 and Table S1, ordered from Sangon Biotech, Shanghai, China): tris (2-carboxyethyl) phosphine hydrochloride, named TCEP solution (Sigma-Aldrich, Shanghai, China); poly-HRP40 (streptavidin modified), named SA-polyHRP (Fitzgerald Industries International Inc., New Castle, DE, USA); TMB Substrate (Neogen, KY, USA); HAuCl_4_ (99.8% Au, Strem Chemicals Inc., Bischheim, France); fetal bovine serum, named FBS (Invitrogen, Carlsbad, CA, USA); all chemical buffer (Sangon Biotech, Shanghai, China). We also used: 16-mutichannel screen-printed carbon electrode, named 16-SPCE (CH Instruments, Inc., Shanghai, China); CHI-660C electrochemical workstation (CH Instruments, Inc., Shanghai, China); NanoDrop One (Thermo Fisher Scientific Inc., Tumwater, WA, USA); Nova nanoSEM 450 instrument (FEI Company, Rockville, MD, USA); and AFM Multimode 8 instrument (Bruker, Billerica, CA, USA).

Human serum samples were derived from healthy human blood, which was donated by volunteers. First, the whole blood was centrifuged at 3500 rpm for 10 min, then the supernatant was collected and kept at 4 °C for later use.

### 2.2. Synthesis and Characterization of TDFs

TDFs of different sizes were synthesized according to the improved process [35]. Briefly, single-stranded A (1 μL), three-thiolated single-stranded B, C, D (1 μL, respectively) and TCEP (10 μL) were added to 86 μL T-buffer (20 mM Tris, 50 mM MgCl_2_, pH 8.0). The mixture was assembled in T100^TM^ PCR Thermal Cycler (heated to 95 °C for 10 min then cooled to 4 °C for 20 min) for later use. The self-assembled TDFs were characterized by 8% native polyacrylamide gel electrophoresis (PAGE), and running condition was 100 V for 120 min in R-buffer (40 mM Tris, 1 mM Ethylene Diamine Tetraacetic Acid (EDTA), pH 8.0), which was visualized under UV light.

### 2.3. Synthesis and Characterization of HCR Structures

Biotin-H1 (10 μM) and biotin-H2 (10 μM) formed hairpin structures, respectively (heated to 95 °C for 10 min, and cooled to 4 °C for 20 min). Then, a mixture containing H1 (1 μM), H2 (1 μM) and different concentrations of initiator (target BRCA-1) were prepared in T-buffer. After that, the mixture was incubated for 2 h at room temperature to form HCR products. The products were characterized by 2% agarose gels (running condition was 150 V for 60 min in R-buffer) and visualized under UV light.

### 2.4. Development of E-cfDNA Sensor

Firstly, the SPCE was pretreated with the electrochemical workstation to generate AuNPs on the working electrode. Before deposition, the SPCE was cleaned by P-buffer (10 mM phosphate buffer, 0.14 M NaCl, 2.7 mM KCl, pH 7.4) and dried by N_2_. Then, 60 μL HAuCl_4_ solution was dribbled onto the electrode surface to form SPGE, the electrodeposition condition was as follows: scan rate, 100 mV/s; deposition time, 300 s; deposition potential, −200 mV. After that, excess HAuCl_4_ solution was removed by P-buffer for later use. Secondly, 10 μL fresh TDF solution (1 μM) was dribbled onto the electrode and incubated at 30 °C over 8 h, and then rinsed by P-buffer. The TDF-modified SPGE was prepared for the following experiments.

### 2.5. cfDNA Detection by E-cfDNA Sensor

Under optimal conditions, the target BRCA-1 was first hybridized with the capture probe (extended chain of TDF) at the SPGE surface in T-buffer for 2 h. At the same time, biotin-H1 (1 μM) and biotin-H2 (1 μM) were heated and annealed as the before condition. After the target BRCA-1 hybridization was completed, H1/H2 mixture (100 nM) was dribbled onto the modified electrode and incubated for 2 h (room temperature). The extra solution was rinsed with P-buffer, and 10 μg/mL SA-polyHRP (3 μL) incubated the electrode surface for 15 min at room temperature. Finally, the electrodes were measured by the CHI-660C, the procedure for testing samples in FBS (50%) and human serum (50%) was the same as above.

## 3. Results and Discussion

### 3.1. Principle of the E-cfDNA Sensor

**Figure 1:**
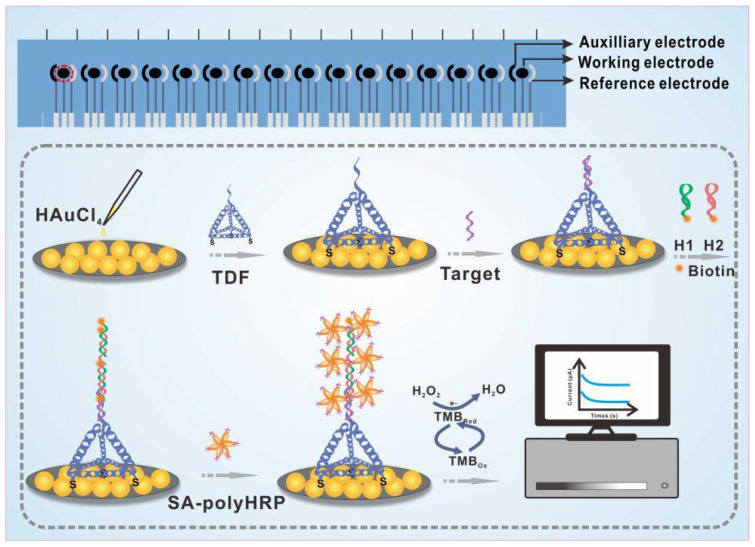
Schematic interpretation of the E-cfDNA sensor. Amperometric current (IT) and cyclic voltammetry (CV) were employed to investigate the performance of this platform.

The design principle of the E-cfDNA sensor for cfDNA detection was based on the redox reaction that convert chemicals signals into electrical signals. As illustrated in Figure 1, firstly, Au NPs were deposited on the surface of the 16-SPCE to form homemade screen-printed gold electrodes (SPGE). Secondly, the TDF contained three thiol group-modified vertices that could be immobilized on the surface of the SPGE through Au-S bonds, and another vertex of the TDF carried a pendant DNA probe that could bind to the target DNA. Thirdly, to further address the limitation of electrochemical biosensors in terms of specificity and sensitivity, we introduced HCR to achieve signal amplification. We used target DNA as the initiator and two biotin-labeled hairpin structures (biotin-H1 and biotin-H2) as the fuel chains. When the target DNA was present, the promoter sequence on target DNA hybridized to H1, forming a cascade reaction to produce HCR products. Finally, SA-polyHRP was attached to the biotin-tagged HCR products and the reduction of H_2_O_2_ was catalyzed in the presence of TMB, resulting in quantitative electrochemical signals.

### 3.2. Characterization and Optimization of Treated Electrode

**Figure 2:**
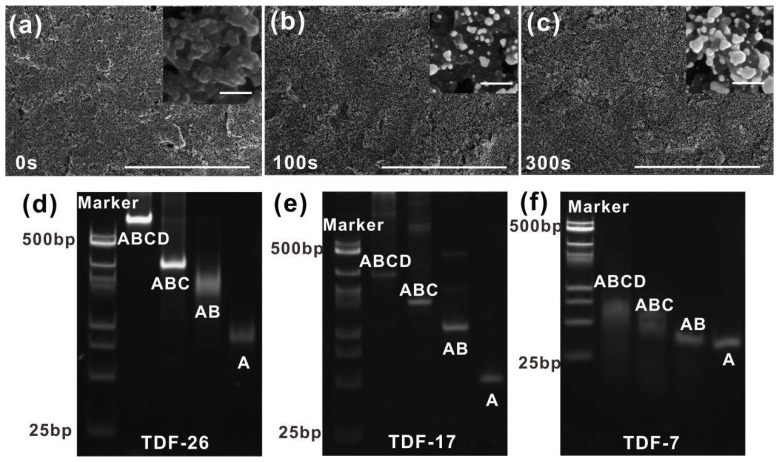
The SEM images of the bare SPCE (**a**) and SPGE with different deposition time: 100 s (**b**), 300 s (**c**). The scale bar was 20 μm. Inserts: Clearer SEM images of SPCE and SPGE. The scale bar was 200 nm. PAGE analysis of the formation of the TDF: (**d**) TDF-26, (**e**) TDF-17 and (**f**) TDF-7.

16-SPCE is easily modified by various nanomaterials (gold, silver, graphene, etc.) to optimize the detection performance [36,37]. Here, we characterized the size and the morphology of the SPCE before and after electrodeposition by SEM. The pristine morphology of the SPCE surface was characterized in Figure 2a. After the bare electrode was deposited, Au NPs were deposited on the electrode surface in an aggregated state (Figure 2b,c). The number and the size of Au NPs on the electrode surface increased with the prolongation of the electrode deposition time, which increased the specific surface area (insert images shown in Figure 2a–c). Clearer SEM images of SPCE and SPGE with different deposition time were provided in Figure S1. The diameter of AuNPs was approximately 123.62 nm, ranging from 56 to 194 nm when the deposition time was 300 s. A larger specific surface area facilitated the subsequent immobilization of the TDFs. To improve the detection performance of SPGE, we optimized the electrodeposition time and the concentration of HAuCl_4_. As shown in Figure S2, the peak current in the CV curve increased with the deposition time and reached the highest value at 300 s, which was taken as the optimal deposition time. Next, the concentration of HAuCl_4_ was optimized using the electrodeposition method. As shown in Figure S3, when the concentration of HAuCl_4_ was 50 μg/mL, the current no longer increased, and we took this concentration as the optimal concentration for preparing electrodes.

In order to subsequently explore the performance of TDFs on the SPGE surface, we first synthesized three different sizes of TDFs: TDF-26, TDF-17 and TDF-7. As shown in Figure 2d–f, native polyacrylamide gel electrophoresis (PAGE) analysis proved that TDFs were successfully assembled independently. Taking TDF-17 as an example, we selected TDF-17, triple-stranded, double-stranded and single-stranded DNA (ABCD, ABC, AB, A) to assemble DNA nanostructures. ABCD shifted slower than ABC, AB and A combinations, proving that the additional sequences and thiol groups did not interfere with the assembly. We also characterized the morphology of TDFs (TDF-17) on the mica surface by using atomic force microscopy (AFM), which indicated that the programmed structure of TDFs with pyramidal configuration was uniform and no aggregates appeared on the surface (Figure S4). The average edge length of the TDF-17 measured was about 6.194 nm, which was close to the theoretical value (Figure S4).

Furthermore, we also investigated the HCR reaction in solution. As shown in Figure S5, in the absence of an initiator (target BRCA-1), biotin-H1 and biotin-H2 maintained hairpin structures when introducing the target BRCA-1 sequence, a partial sequence of this target hybridized to the biotin-H1 strand, and the HCR reaction was triggered by alternately adding biotin-H1 and biotin-H2 to form long HCR products. It has been previously reported that the length of HCR products are inversely proportional to the concentration of the initiator. When the concentration of the target BRCA-1 was 1 μM, the length of the product was about 500 bp. When the concentration of the target BRCA-1 was 0.1 μM, the length of the product was about 1000 bp. Later, these long HCR products could bind more SA-polyHRP, ultimately achieving efficient signal amplification.

### 3.3. Comparison of Capture Performance among Different Probes

**Figure 3:**
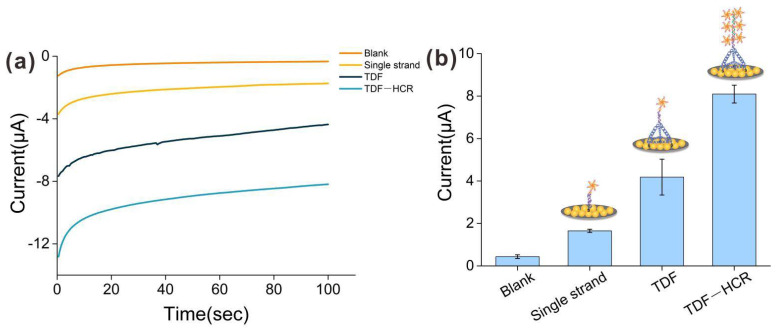
(**a**) Typical I-T curves for three kinds of probes (single strand group, TDF group and TDF-HCR group) modified on SPGE at target concentration of 1 nM. The potential was held at 100 mV and the reduction current was recorded at 100 s. (**b**) The corresponding current of three kinds of probes when the scan time was 100 s. Error bars represent the SD of at least 3 independent experiments.

To evaluate the capture performance of the TDF-HCR-based E-cfDNA sensor, we first employed TDF-17 as the capture probe. The single-strand group and the single-TDF group were designed as controls. As shown in Figure 3a, the amperometry was used to directly characterize the electrochemical process of different probes. At an initial potential of 100 mV, we immediately obtained an attenuation curve of current (I) versus time (t), which leveled out within 100 s. As shown in Figure 3b, the blank current of the E-cfDNA sensor was as low as 0.33 μA, demonstrating little non-specific adsorption of nucleic acid or enzymes. The current was 1.65 μA for the single-strand group and 4.18 μA for single-TDF group, while the current was 8.09 μA for TDF-HCR group. The same trend appeared in the cyclic voltammetry (CV) tests (Figure S6). Compared with the single-stranded DNA capture probe, three-dimensional TDF as a rigid scaffold could be anchored on the surface of gold electrodes, and the extended strand at the vertex of the TDF maintained an ordered and upright orientation. In addition, TDF has a spatial structure that can enlarge the distance between probes, avoiding intermolecular entanglement. Furthermore, compared with the single-TDF group, the TDF-HCR strategy showed more than two times higher than the current signals. This result was attributed to the target DNA initiating the cascade HCR to form a long product, and the biotin-labeled product provided numerous binding sites for binding multiple avidin-labeled polyHRP, resulting in a significant increase in signal.

### 3.4. Performance Verification of the E-cfDNA Sensor Mediated by TDF Regulation

**Figure 4:**
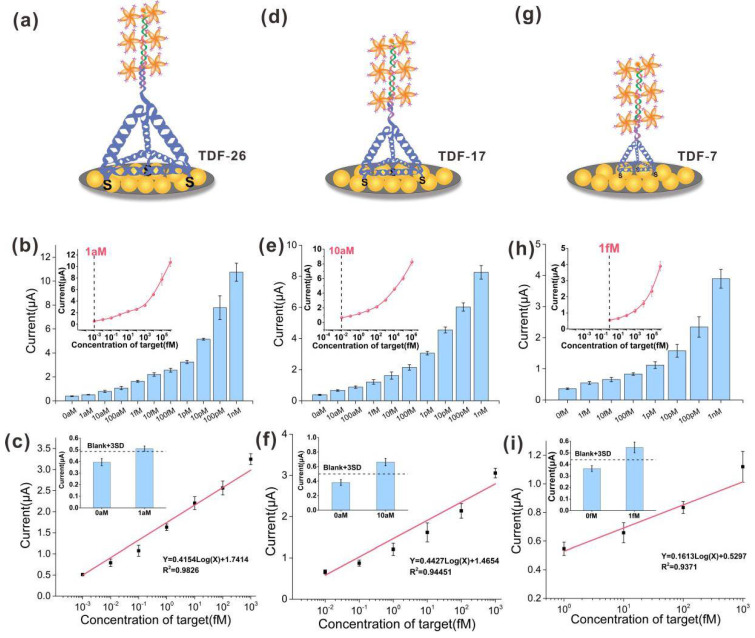
Sensitivity of the E-cfDNA sensor mediated by differently sized TDF (TDF-26, TDF-17, TDF-7). (**a**,**d**,**g**) Scheme illustration. (**b**,**e**,**h**) Amperometric current amplification with corresponding increased concentration (from 0 nM to 1 nM) of target DNA. Insert: a dose–response curve between DNA concentration and current. (**c**,**f**,**i**) Linear calibration curves. Insert: Limits of detection. Error bars represent the SD of at least 3 independent experiments.

Modulating the orientation and density of capture probes can improve hybridization efficiency between the target DNA and probe [38,39]. Here, we designed and programmed differently sized TDFs to accurately modulate the density of the capture probes on the SPGE surface (Figure 4a,d,g). Three sizes of TDFs were used: TDF-26, TDF-17 and TDF-7, each of which contained 26, 17, and 7 base pairs on each edge, and the corresponding theoretically calculated edge lengths were 8.8 nm, 5.8 nm, 2.4 nm, respectively. As shown in Figure 4, the response current signal increased significantly as the concentration of the target BRCA-1 increased from 0 nM to 1 nM, which suggested that the electrochemical signal closely depended on the concentration of the target (Figure 4b,e,h). However, each group had different detection limits and linear ranges for the target. For the DTF-26 group (Figure 4c), the linear detection ranged from 1 aM to 1 pM with a 1 aM limit of detection (LOD), and the regression equation was Y = 0.4154 Log(X) + 1.7414 (R^2^ = 0.9826). For the DTSP-17 group (Figure 4f), the linear detection ranged from 10 aM to 1 pM with a 10 aM LOD, the regression equation was Y = 0.4427Log(X) + 1.4654 (R^2^ = 0.9445). For the DTSP-17 group (Figure 4i), the linear detection ranged from 1 fM to 1 pM with a 1 fM LOD, and the regression equation was Y = 0.1613Log(X) + 0.5294 (R^2^ = 0.9371). Based on the above results, we demonstrated that as the size of the TDFs increased, the concentration of the lowest detectable target molecule decreased, and the sensitivity increased. In previously reported studies, Lin et al. found that the assembly density of DNA tetrahedral probes was inversely proportional to their size, and the hybridization efficiency of probes on the interface also heavily depended on the distance between probes [26]. That is, within a certain range, the larger the size of the DNA tetrahedron, the longer distance between the probes, resulting in a higher hybridization efficiency of the probes. In this work, due to the jointly optimized Au deposition substrate and HCR amplification system, the lowest detection limit can be as low as 1 aM, which is far superior to previous reports (Table 2).

### 3.5. Selective Ability of the E-cfDNA Sensor

**Figure 5:**
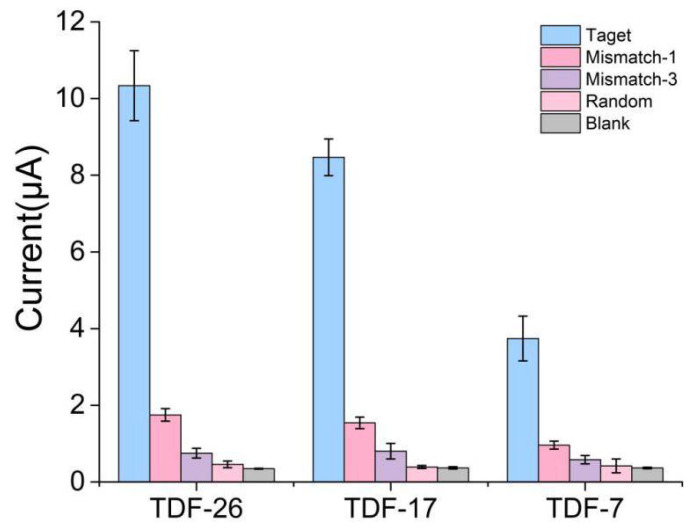
Specificity investigation of differently sized TDFs for target DNA (1 nM) and other mismatch DNA (1 nM). Error bars represent the SD of at least 3 independent experiments.

The selectivity and specificity of the proposed E-cfDNA sensor was tested by using a perfectly matched sequence, a single-base mismatch DNA sequence (Mismatch-1), a three-base mismatched DNA sequence (Mismatch-3), and a random DNA sequence (Random) as detection targets (Table 1). As shown in Figure 5, the three types of TDF/HCR-based sensors could easily distinguish the target sequence from the mismatched sequence. Taking TDF-26 as an example, the amperometric current corresponding to the perfectly matched target (1 nM) was 10.335 μA, whereas the amperometric current corresponding to Mismatch-1, Mismatch-3 and Random were 1.749 μA, 0.751 μA, 0.459 μA, respectively. The current signals corresponding to target DNA were significantly higher than that of mismatched DNA, up to 22-fold, demonstrating the high specificity of the sensor. The results were mainly due to the uniqueness of the design, as shown in Figure S1. HCR cannot form a cascade reaction without the target sequence.

### 3.6. Application to the Clinical Utility

**Figure 6:**
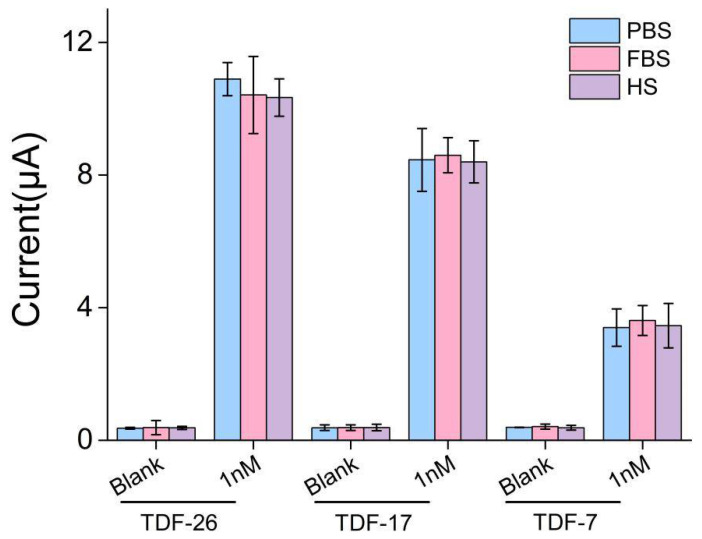
Performance verification of E-cfDNA sensor in PBS buffer, 50% fetal bovine serum (FBS) and 50% human serum (HS). Error bars represent the SD of at least 3 independent experiments.

To further demonstrate the superiority of our electrochemical biosensor, the sensor was used to detect cell-free DNA in serum to demonstrate capture performance in complex components. We spiked the 1 nM of target BRCA-1 into fetal bovine serum (50%) and human serum (50%). As shown in Figure 6, the functionalized electrodes exhibited negligible signal changes (less than 7%) in either fetal bovine serum or human serum compared with PBS buffer, which indicated that the possibility of the E-cfDNA sensor can be used in clinical samples.

## 4. Conclusions

In conclusion, we fabricated a home-made AuNP-deposited multi-channel electrode, and then exploited the ultrasensitive E-cfDNA sensor for specific cell-free DNA detection based on the programmed TDF and HCR. The TDF nanostructures immobilized on the electrode interface served as rigid scaffolds, and hybridized with the target sequence to trigger the HCR reaction, which achieved signal amplification. Compared to the traditional single-stranded capture probe, our TDF-HCR strategy showed over eight-fold increased amperometric current signals for detection of the target BRCA-1 gene. To further improve the sensitivity of the E-cfDNA sensor, we programmed TDFs of different sizes to precisely control the orientation of the capture probes and the distance probe-to-probe. Each of the TDF group exhibited a linear response to its target DNA, especially TDF-26, which showed the highest amperometric current signals with an ultra-low detection limit of 1 aM. In addition, our E-cfDNA sensor also maintained ultrahigh sensitivity in complex matrices, revealing promise for clinical early tumor detection, mutation screening and prognosis.

## Figures and Tables

**Table 1:** Nucleotide sequences for selective ability of the E-cfDNA sensor.

Name	Sequence (5′-3′)
Mismatch-1	TGGTAACAGTGTGAGGTTTAACG GAACAAA T GGAAGAAAATC
Mismatch-3	TGGTAACAGTGTGAGGTTTAACG GAACAA G A T GA T GAAAATC
Random	TGGTAACAGTGTGAGGTTTAACG TCGATGCCTGATCTTGGTA
Target-BRCA-1	TGGTAACAGTGTGAGGTTTAACG GAACAAAAGGAAGAAAATC

**Table 2:** Comparison of the E-cfDNA sensor with other techniques for DNA detection.

Techniques	Name	Linear Range	LOD	Refs.
Fluorescence Biosensors	CRISPR-Cas12a-based cfDNA biosensing system	1 fM to 100 pM	0.34 fM	[8]
	DNA tetrahedral-based fluorescent microarray platform	100 aM to 1 pM	10 aM	[39]
Electrochemical Biosensors	Label-free electrochemical biosensor	0.01 fM to1 pM	2.4 aM	[28]
	HCR and DNA nanostructure-based electrochemical biosensor	1 fM to 100 pM	100 aM	[34]
Electrochemiluminescence Biosensors	DNA walk-based electrochemiluminescence biosensing	1 fM to 100 pM	0.18 fM	[40]
	Cas12a-based electrochemiluminescence biosensor	1 pM to 10 nM	0.48 pM	[41]
E-cfDNA sensor		1 aM to 1 pM	1 aM	This work

## Data Availability

The data is available on reasonable request from the corresponding author.

## Supplementary Materials

The following are available online at https://www.mdpi.com/article/10.3390/nano12040666/s1: Table S1: DNA Sequences used in the work; Figure S1: The SEM images of AuNPs deposited on SPGE; Figure S2: Optimizing the deposition time of HAuCl_4_ on SPCE; Figure S3: Optimizing the deposition concentration of HAuCl_4_ on SPCE; Figure S4: The AFM image of TDF-17; Figure S5: Characterization of the HCR products; Figure S6: Cyclic voltammetry (CV) of different capture probes.
