# Supplementary material for: Tetrahedral DNA Framework-Programmed Electrochemical Biosenors with Gold Nanoparticles for Ultrasensitive Cell-Free DNA Detection
Source: Nanomaterials (Basel). 2022 Feb 16;12(4):666. doi: 10.3390/nano12040666 (PMC8879424; doi:10.3390/nano12040666)
Supplement: Supplementary file 1 [file nanomaterials-12-00666-s001.zip › nanomaterials-1578527-supplementary.pdf]

nanomaterials

MDPI

Tetrahedral DNA Framework-Programmed Electrochemical Biosenors with Gold Nanoparticles for Ultrasensitive Cell-Free DNA Detection

Chenguang Wang [1,2,†], Wei Wang [3,†], Yi Xu [1], Xiaoshuang Zhao [4], Shuainai Li [1,2], Qiuling Qian [1,2] and Xianqiang Mi [1,2,4,5,6,*]

1 Shanghai Advanced Research Institute, Chinese Academy of Sciences, Shanghai 201210, China; wangcg@sari.ac.cn (C.W.); xuyi@sari.ac.cn (Y.X.); lishuainan2019@sari.ac.cn (S.L.); qianqiuling2018@sari.ac.cn (Q.L.)
2 University of Chinese Academy of Sciences, Beijing 100049, China
3 Shanghai Pudong New District Zhoupu Hospital (Shanghai University of Medicine & Health Sciences Affiliated Zhoupu Hospital), Shanghai 201318, China; doctorww1978@163.com
4 State Key Laboratory of Functional Material for Informatics, Shanghai Institute of Microsystem and Information Technology, Chinese Academy of Sciences, Shanghai 200050, China; zxsh1217@mail.sim.ac.cn
5 CAS Center for Excellence in Superconducting Electronics (CENSE), Shanghai 200050, China
6 Key Laboratory of Systems Health Science of Zhejiang Province, Hangzhou Institute for Advanced Study, University of Chinese Academy of Sciences, Chinese Academy of Sciences, Hangzhou 310024, China
* Correspondence: mixq@mail.sim.ac.cn
† These authors contributed equally to this work.

Table S1. Sequences for oligonucleotide used in this work.

Name	Sequence (5′-3′)
A-7-capture	GAGCGTTAGCCACACACACAGTCTTTTTTTTTTGATTTTCTTCCTTTTGTTC
B-7	SH-C6-TTAGGCGAGTGTGGCAGAGGTGT
C-7	SH-C6-CGCCTAAACAAGTGGAGACTGTG
D-7	SH-C6-AACGCTCACCACTTGAACACCTC
A-17-capture	ACATTCCTAAGTCTGAAACATTACAGCTTGCTACACGAGAAGAGCCGCCATAGTATTTTTTTTTTG ATTTTCTTCCTTTTGTTC
B-17	SH-C6-TATCACCAGGCAGTTGACAGTGTAGCAAGCTGTAATAGATGCGAGGGTCCAATAC
C-17	SH-C6-TCAACTGCCTGGTGATAAAACGACACTACGTGGGAATCTACTATGGCGGCTCTTC
D-17	SH-C6-TTCAGACTTAGGAATGTGCTTCCCACGTAGTGTCGTTTGTATTGGACCCTCGCAT
A-26-capture	GCCTGGAGATACATGCACATTACGGCTTTCCCTATTAGAAGGTCTCAGGTGCGCGTTTCGGTAAG TAGACGGGACCAGTTCGCCATTTTTTTTTTGATTTTCTTCCTTTTGTTC
B-26	SH-C6- CGCGCACCTGAGACCTTCTAATAGGGTTTGCGACAGTCGTTCAACTAGAATGCCCTTTGGGCTGTT CCGGGTGTGGCTCGTCGG
C-26	SH-C6- GGCCGAGGACTCCTGCTCCGCTGCGGTTTGGCGAACTGGTCCCGTCTACTTACCGTTTCCGACGA GCCACACCCGGAACAGCCC
D-26	SH-C6- GCCGTAATGTGCATGTATCTCCAGGCTTTCCGCAGCGGAGCAGGAGTCCTCGGCCTTTGGGCATT CTAGTTGAACGACTGTCGC
Single capturer	SH-C6-GAGCGTTAGCTTTTTTTTTTGATTTTCTTCCTTTTGTTC
Target-BRCA-1	TGGTAACAGTGTGAGGTTTAACGGAACAAAAGGAAGAAAATC
BRCA-1-biotin	Biotin-GAACAAAAGGAAGAAAATC
H1-biotin	Biotin-CGTTAAACCTCACACTGTTACCAAGTTTGTGGTAACAGTGTGAGGT
H2-biotin	Biotin-TGGTAACAGTGTGAGGTTTAACGACCTCACACTGTTACCACAAACT

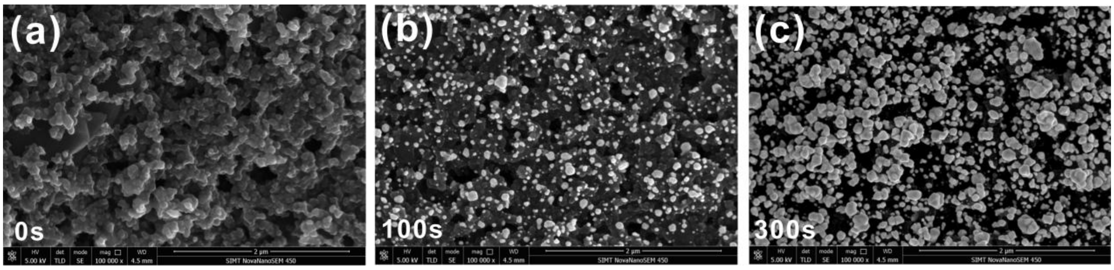

Figure S1. The SEM images of the bare SPCE (**a**) and SPGE with different deposition time: 100 s (**b**), 300 s (**c**). The scale value was 2 μm.

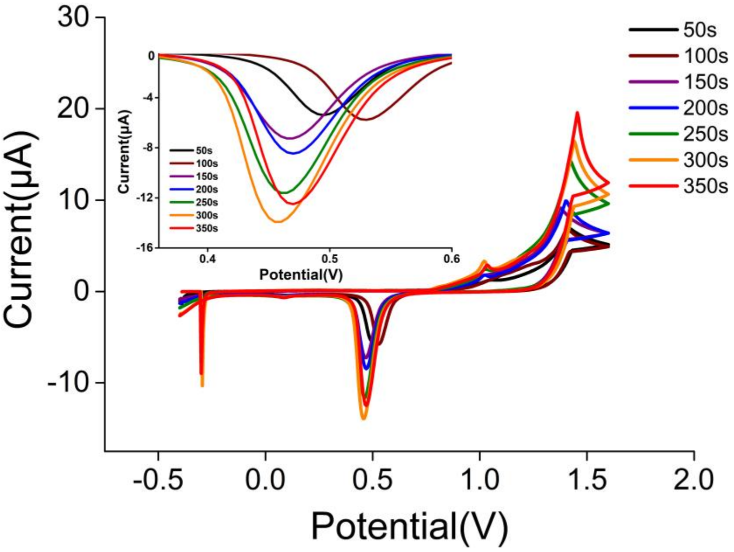

Figure S2. Investigation on the deposition time of $HAuCl_4$. The integral CVs results recorded for the Au NPs modified 16-SPCE in 0.5 M H_2SO_4, scan rate: 100 mV/s.

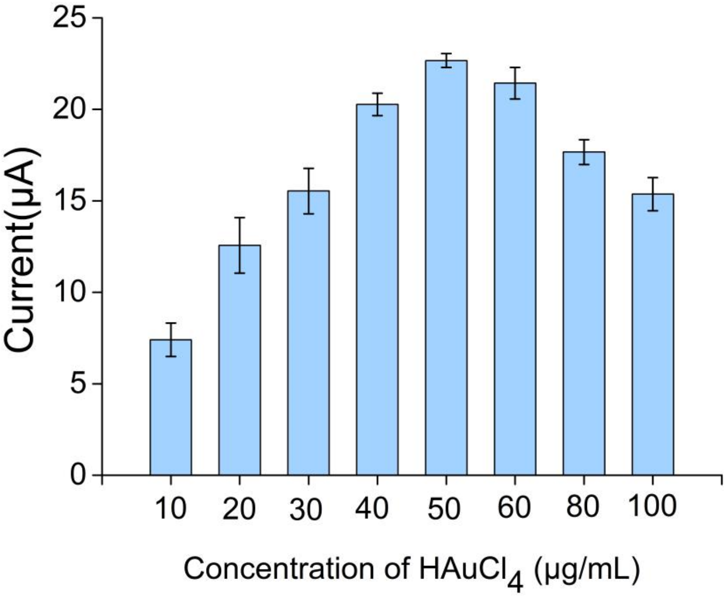

Figure S3. Investigation on the concentration of $HAuCl_4$. CVs results responsed for 10, 20, 30, 40, 50, 60, 80, 100 μg/mL $HAuCl_4$ solution. The Au NPs modified 16-SPCE was scanned in 0.5 M H_2SO_4.

The electrodeposition time was 300 s. Error bars represented the SD of at least 3 independent experiments.

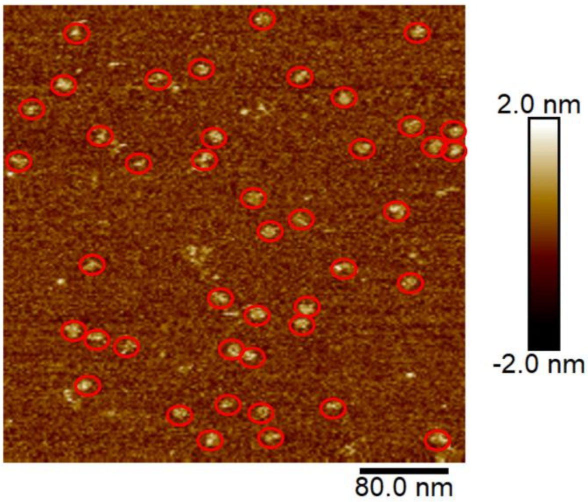

Figure S4. The structure characterization of TDF-17 by AFM. The average edge length of the TDF-17 measured was about 6.194 nm, which was close to the theoretical value (5.8 nm). Scale bar = 80nm.

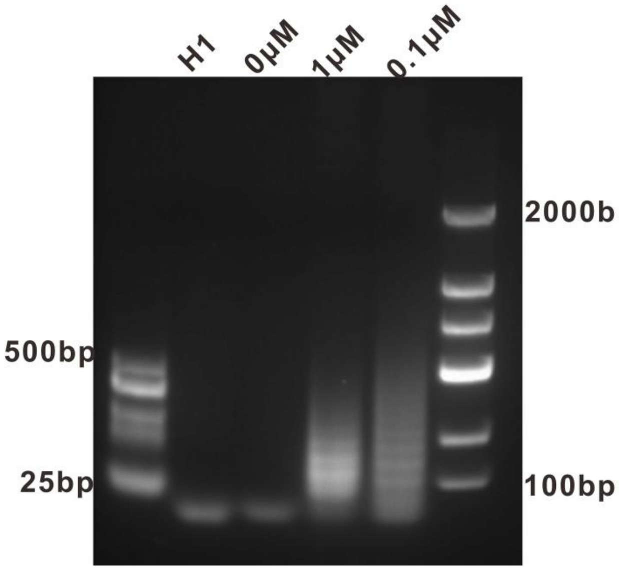

Figure S5. Agarose gel electrophoresis image analysis for HCR. From left to right, lane1 and lane6, 20-bp and 2000-bp DNA ladder markers, respectively; Lane2, H1; Lane3, H1 + H2, without initiator; Lane4, H1 + H2 + initiator (1 μM); Lane5, H1+H2+initiator (0.1 μM).

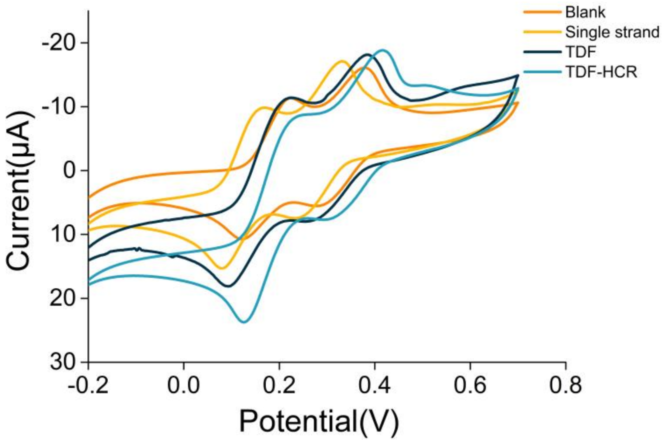

Figure S6. Typical CV curves for different probes modified on SPGE at target concentration of 1 nM. It conducted at a scanning rate of 0.1 V/s ranging from –0.3 V to 0.7 V.
